# Quantitative Recovery of Viable *Lactobacillus paracasei* CNCM I-1572 (L. casei DG®) After Gastrointestinal Passage in Healthy Adults

**DOI:** 10.3389/fmicb.2018.01720

**Published:** 2018-08-02

**Authors:** Stefania Arioli, Ranjan Koirala, Valentina Taverniti, Walter Fiore, Simone Guglielmetti

**Affiliations:** ^1^Division of Food Microbiology and Bioprocesses, Department of Food, Environmental and Nutritional Sciences (DeFENS), University of Milan, Milan, Italy; ^2^Sofar S.p.A., Trezzano Rosa, Italy

**Keywords:** probiotic, Enterolactis, EPS, qPCR, isolation

## Abstract

Probiotics are live microorganisms, and viability after transit through the gastrointestinal tract (GIT) is considered an inherent property of the health benefits of probiotics. The aim of the present study was to quantify the viable and total loads of *Lactobacillus paracasei* DG cells after passage through the GIT following the consumption of the probiotic product Enterolactis (L. casei DG®; *L. paracasei* CNCM I-1572; *L. paracasei* DG) from drinkable vials by healthy adults. We developed a novel method for discriminating and enumerating culturable *L. paracasei* DG cells based on the unique sticky, filamentous phenotype of this strain on MRS agar containing vancomycin and kanamycin. The identity of DG was also confirmed with strain-specific primers by colony PCR. This method was used for a recovery study of the DG strain to quantify viable cells in the fecal samples of 20 volunteers during a 1-week probiotic consumption period and a 1-week follow-up. We isolated *L. paracasei* DG from at least one fecal sample from all the volunteers. The highest concentration of viable DG cells [ranging from 3.6 to 6.7 log_10_ colony-forming unit (CFU) per gram of feces] in the feces was observed between 4 and 8 days from the beginning of Enterolactis intake and for up to 5 days after cessation of intake. As expected, the total DG count determined by real-time quantitative PCR (qPCR) was mostly higher than the viable DG cells recovered. Viable count experiments, carried out by combining *ad hoc* culture-based discriminative conditions and strain-specific molecular biological protocols, unambiguously demonstrated that *L. paracasei* DG can survive gastrointestinal transit in healthy adults when ingested as Enterolactis in drinkable vials containing no less than one billion CFU at the end of shelf life.

## Introduction

Probiotics are “live microorganisms that, when administered in adequate amounts, confer a health benefit on the host” (Hill et al., 2014). Therefore, by definition, the term probiotic is restricted to live microbial cells. According to the regulations of numerous countries, the actual number of microbial colony-forming units (CFUs) in a probiotic product cannot be lower than the value indicated on the label until the end of the shelf life of the product. Consequently, both producers and competent public authorities constantly assess the viable counts of commercial probiotic products to ensure compliance with label specifications. Simultaneously, many industrial efforts are being made to identify strategies to keep bacterial cells viable during the various production steps and in the final product until the end of the shelf life; these strategies include the selection of appropriate culture media, the use of protective agents during the freeze-drying process, microencapsulation, and improvements in packaging systems (da Cruz et al., 2007; Savini et al., 2010; Goderska, 2012; Mai et al., 2017).

Although microbial cell viability is constantly monitored for each marketed probiotic product, only limited data are available regarding the capacity of a particular microbial strain in a specific probiotic formulation to survive in the gastrointestinal tract (GIT) upon ingestion. Nonetheless, viability is conventionally considered a prerequisite for the health benefit of a probiotic, and accordingly, viable probiotics have been demonstrated to be more effective than non-viable probiotics for certain health-promoting activities (Lahtinen, 2012). In this context, the first “FAO/WHO Expert Consultation on Evaluation of Health and Nutritional Properties of Probiotics” stated that the ability to remain viable at the target site should be verified for each potential strain (FAO/WHO, 2001).

The conventional process of selecting novel potential probiotic strains includes *in vitro* assessment of the ability of the strains to survive at low pH, in simulated gastric juice or in the presence of bile salts. However, *in vivo* assessment of probiotic viability is a more challenging task, possibly due to the difficulties associated with setting up intervention trials with human volunteers and because of technical limitations. The use of human biopsies is an impractical option, and therefore, the ability of probiotic microorganisms to survive in the GIT is assessed by analyzing fecal samples. However, conventional selective and/or discriminative growth media can barely distinguish a specific probiotic strain from other members of closely related taxa that are naturally present in the sample. The development of molecular approaches based on strain-specific primer design may solve the problem of selectivity, although PCR protocols may lack sensitivity and, most importantly, will not permit assessment of the viability of the probiotic cells. For these reasons, culture-based methods have been combined with molecular approaches to obtain adequate sensitivity and specificity (Dommels et al., 2009; Poutsiaka et al., 2017). Nevertheless, *in vivo* assessment of the ability to survive gastrointestinal transit has been carried out so far for only a limited number of well-known commercial probiotics (Verna and Lucak, 2010; Derrien and van Hylckama Vlieg, 2015).

*Lactobacillus paracasei* CNCM I-1572 (commercially known as L. casei DG®; *L. paracasei* DG) is a bacterial strain commercially available as part of the Enterolactis® product line. Enterolactis is currently the best-selling probiotic food supplement in Italy, which is the country with the largest probiotic market in the world. *L. paracasei* DG has been demonstrated to possess the ability to modulate the intestinal microbial ecosystems of healthy adults (Ferrario et al., 2014) and to influence host immune response (Balzaretti et al., 2015; Cremon et al., 2017) via its unique polysaccharide capsule (Balzaretti et al., 2017). *L. paracasei* DG has also been demonstrated to possess therapeutic potential for several dysfunctions and pathological conditions such as ulcerative colitis (D’Incà et al., 2011), diverticular disease (Tursi et al., 2013; Turco et al., 2017), small intestinal bacterial overgrowth (Rosania et al., 2013), and irritable bowel syndrome (Compare et al., 2017; Cremon et al., 2017).

In this study, we present the development of a strategy that combines culture-based methods and molecular methods for strain-specific selective enumeration of viable *L. paracasei* DG cells in fecal samples. Subsequently, we adopted this protocol to demonstrate the ability of *L. paracasei* DG to survive gastrointestinal transit when consumed by healthy adults via a probiotic formulation consisting of at least one billion bacterial CFU in a 10-ml suspension.

## Materials and Methods

### Bacterial Strain and Selective Medium

*Lactobacillus paracasei* DG (CNCM I-1572) was routinely cultivated anaerobically at 37°C for 24 h in MRS broth or in vk-MRS agar (Difco Laboratories Inc., Detroit, MI, United States) supplemented with 1 μg/ml vancomycin and 10 μg/ml kanamycin (Sigma-Aldrich, Steinheim, Germany). The culturable bacterial content per vial used for the study was determined by resuspending at least 5 g of freeze-dried *L. paracasei* DG biomass in maximum recovery diluent (MRD) (Scharlab, Milan, Italy); then, this initial cell suspension was homogenized in a sterile Stomacher bag by using a Colworth Stomacher 400 instrument (Seward, West Sussex, United Kingdom) for 3 min. Serial 10-fold dilutions were prepared in MRD, and total microorganismal content was determined by the spread plate technique on vk-MRS agar.

### Human Intervention Study Methods

**FIGURE 1:**
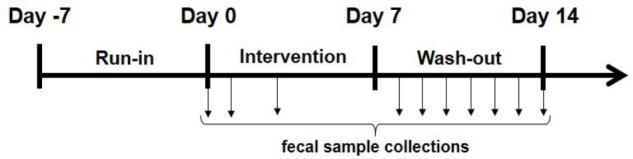
Design of the study. Vertical arrows indicate the day of collection of the fecal sample (when available) from the volunteer.

*Study title*: recovery study with L. casei DG® (Enterolactis®) in drinkable vials in healthy adult volunteers (REVENANT-DG). *Study design*: open-label pilot microbiological study (**Figure 1**). *Number of participants*: 20 volunteers (**Table 1**). *Study population*: healthy (non-diseased) adult volunteers of both sexes, aged 18–55 years, who provided signed informed consent of their participation in the study. Exclusion criteria were as follows: (i) antibiotic consumption in the month preceding the start of the trial; (ii) consumption of antacids or prokinetic gastrointestinal drugs; (iii) chronic inflammatory bowel diseases; (iv) intestinal diseases of infectious origin; (v) episodes of viral or bacterial enteritis in the 2 months prior to the study; (vi) episodes of gastric or duodenal ulcers in the previous 5 years; (vii) pregnancy or breast-feeding; (viii) recent history of alcohol abuse or suspected drug use; and (ix) any severe disease that may interfere with treatment. *Probiotic formulation under study*: Enterolactis® (Sofar, Trezzano Rosa, Italy) in drinkable vials, which consisted of a plastic vial containing 10 ml of 2% fructose solution (additives: citric acid as an acidity controller, and sodium benzoate and potassium benzoate as preservatives) and a plastic/aluminum push-button cap (DryCap technology) containing at least one billion CFU/vial of freeze-dried *L. paracasei* DG biomass. *Study protocol*: during the initial visit, each volunteer provided signed informed consent and was trained on the entire procedure; then, the study consisted of a pre-recruitment phase (run-in, 1 week), during which the volunteers followed their conventional diet with a ban on probiotic-fermented milks (traditional yogurt was allowed during this phase) and probiotic, prebiotic and symbiotic foods and supplements. At the end of this period, the volunteers were invited to consume one drinkable vial of Enterolactis per day for 1 week. The product was consumed on an empty stomach in the morning, at least 10 min before breakfast, or, if forgotten, in the evening, before bedtime and at least 2 h after the last meal. Following the 7 days of administration, the volunteers underwent a 1-week follow-up, which was identical to the period of pre-recruitment. *Sample collection*: at the beginning of the study, the volunteers were trained to collect and deliver the fecal samples as follows: each stool specimen (at least 2 g) was collected in special sterile containers, stored at room temperature, and delivered to the laboratory within 24 h. Preliminary experiments demonstrated that strain DG can survive in human feces at room temperature and 37°C at least 48 h without significant decrease of the viable count (not shown). To verify the ability of the DG strain to survive passage through the GIT, the fecal samples collected were immediately subjected to viable bacterial counts. To obtain fecal bacterial counts, 1-g fecal samples were diluted in MRD, homogenized in a sterile Stomacher bag, plated on vk-MRS and incubated anaerobically at 37°C for 48 h. Throughout the study period, the frequency and consistency of the stools were evaluated according to a validated fecal scoring system (Bristol stool scale). *Ethical statement*: the study protocol was approved by the Research Ethics Committee of the Università degli Studi di Milano (opinion no. 37/16, 15th December 2016). Written informed consent was obtained from all the subjects before recruitment. *Volunteer compliance*: volunteer compliance, as determined by verbal assessment, was almost 100%. All programmed fecal samples were delivered by volunteers, with the only exception of subject S1, we voluntarily interrupted fecal sample collections at day 12.

**Table 1:** Basic characteristics of study participants.

Subject (*n* = 20)	Sex (8F/12M)	Age (22–53 years)
S01	M	53
S02	F	46
S03	F	47
S04	F	27
S05	F	27
S06	F	28
S07	M	25
S08	F	23
S09	M	23
S10	F	27
S11	M	24
S12	M	28
S13	F	31
S14	M	31
S15	M	31
S16	F	26
S17	M	26
S18	F	26
S19	F	42
S20	M	22

### DNA Extraction

After microbiological analysis, the fecal samples were stored at −80°C until DNA extraction. Samples collected from each subject on different days (*n* = 9; T0–T8) were thawed on ice and mixed vigorously for 2–3 min with a sterile spatula; then, 250 mg of each sample was weighed and processed with a DNeasy® PowerLyzer® PowerSoil® Kit (Qiagen, Hilden, Germany) with the following modifications: tubes containing samples were incubated at 65°C for 10 min after addition of solution C1. Before extraction, mechanical lysis of the cells was carried out using a Precellys 24 bead homogenizer (Bertin Technologies, Montigny le Bretonneux, France). Then, the extraction was conducted according to the manufacturer’s specifications. The DNA extracted from fecal samples was quantified by using a NanoDrop (BioTek Instruments, Inc., CA, United States). Finally, the DNA was stored at −80°C until molecular analysis.

### *L. paracasei* DG Quantification by qPCR

Real-time quantitative PCR (qPCR) protocols were adopted for the quantification of *L. paracasei* DG in fecal metagenomic DNA, targeting the glycosyl transferase gene *welF* with the primers rtWELFf (5′-TACTAAAGAAATTAGCTTTTGT-3′) and rtWELFr (5′-AGTAATGTCTGCATCCTCCA-3′) (Ferrario et al., 2014) in a final volume of 15 μl containing 7.5 μl of EvaGreen® Supermix (Bio-Rad Laboratories, Segrate, Italy) and 0.5 μM of each primer; 50 ng of template DNA samples was used in each reaction. The amplification was carried out using the following thermal program: initial hold at 95°C for 3 min followed by 39 cycles of 95°C for 30 s, 58°C for 30 s, and 72°C for 30 s. A standard calibration curve for the absolute quantification of the total number of L. casei DG® was prepared by mixing five different fecal samples (of varying consistency) that were collected before the consumption of probiotics. Different numbers of L. casei DG® cells (*n* = 10; 1–1 × 10^9^) were added to 250-mg fecal samples; one fecal sample was used as a control (without the addition of bacterial cells). All the samples were subjected to DNA extraction as mentioned above. The standard curve was obtained by plotting the average *C*_q_ values versus log_10_ of the number of cells added to each fecal sample. Melting curves were analyzed with Bio-Rad CFX Manager 3.1 software to confirm the specificity of the amplification products.

### Colony PCR for Identification of *L. paracasei* DG Colonies

To confirm the identities of the DG colonies, we carried out end-point colony PCR by randomly selecting colonies with sticky, filamentous phenotypes. Colonies with different phenotypes were always included as negative controls. PCRs were performed in 25-μl reaction mixtures, each containing one colony (picked with a sterile wooden stick), 2.5 μl of 10× reaction buffer, 200 μmol/l of each dNTP, 0.5 mmol/l MgCl_2_, 0.5 μmol/l each primer (rtWELFf and rtWELFr), and 0.5 U DreamTaqTM DNA polymerase (Thermo Fisher Scientific Inc., Monza, Italy). Amplifications were carried out using a Mastercycler 96 (Eppendorf, Milan, Italy). The PCR mixtures were subjected to the following thermal cycling conditions: initial hold at 95°C for 3 min followed by 39 cycles of 95°C for 30 s, 58°C for 30 s, and 72°C for 30 s. Amplification products were resolved by electrophoresis on a 2% (w/v) agarose gel (with 0.2 μg/ml ethidium bromide) in 1× TAE buffer (40 mmol/l Tris-acetate, 1 mmol/l EDTA, pH 8.0) and photographed. A 1-kb GeneRuler DNA Ladder Mix was used as a size marker.

## Results

### Development of a Method for the Enumeration of Live *L. paracasei* DG Cells

**FIGURE 2:**
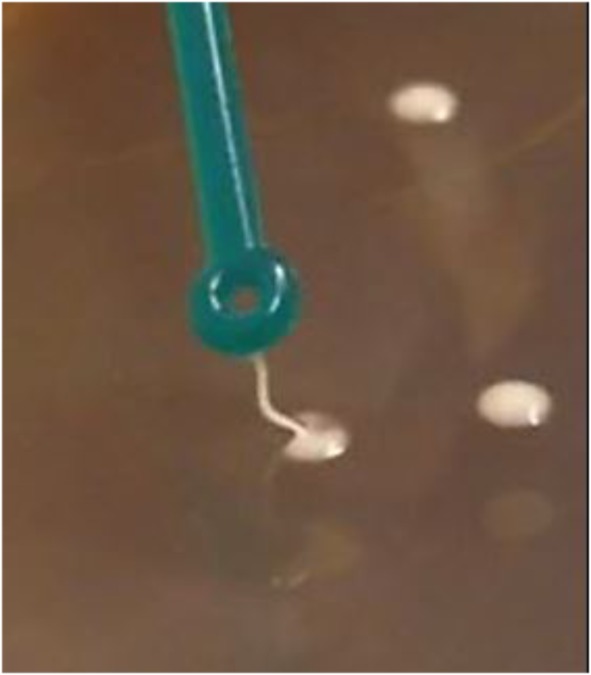
Sticky, filamentous consistency of the colonies of *Lactobacillus paracasei* DG grown on vk-MRS agar plates.

To develop culture conditions for selective and discriminative growth of *L. paracasei* DG, we implemented the cultivation protocol suggested by the Italian Higher Institute of Health (ISS) for the enumeration of heterofermentative lactobacilli in probiotic products (ISSN 1123-3117 ISTISAN 08/36; available at http://www.iss.it/binary/publ/cont/08-36_web.1229959899.pdf as accessed on February 10th, 2018). The ISSN protocol suggested the use of 1 μg/ml vancomycin for the selective counting of heterofermentative lactobacilli; however, during the preliminary experiment, the use of such a medium allowed the growth of many non-DG colonies, which hampered the identification and counting of the probiotic strain under investigation. For this reason, based on the antibiotic resistance profile of *L. paracasei* DG (**Table 2**), we also added 10 μg/ml kanamycin to the medium (vk-MRS medium), which resulted in an evident decrease in the background without affecting the growth of the DG strain compared to the growth of this strain in normal MRS medium or MRS supplemented with the only vancomycin. The use of higher concentrations of the antibiotics resulted in reduced viable count of the DG strain. In addition, we observed that the DG colonies on vk-MRS agar had a peculiar sticky, filamentous phenotype, allowing the discrimination of this strain from the colonies of closely related lactobacilli, which typically have a creamy consistency (**Figure 2** and Supplementary File S1).

**Table 2:** Minimum inhibitory concentrations (MICs) of *Lactobacillus paracasei* DG and the EFSA reference strain *L. paracasei* LMG 12586 determined via a microdilution assay.

Antibiotic molecule	ISO10932 (μg/ml)^∗^	MIC (μg/ml) LMG 12586	MIC (μg/ml) *L. paracasei* DG
Ampicillin	0.5–2	1	1
Vancomycin	Not required	>16	>16
Gentamycin	1–4	4	4
Kanamycin	16–64	32	256
Streptomycin	8–32	16	32
Erythromycin	0.062–0.25	0.125	0.125
Clindamycin	0.062–0.25	0.125	0.125
Tetracycline	1–4	2	8
Chloramphenicol	4–8	4	4

*The results are compared with cutoff values defined by EFSA (^∗^) for the purpose of distinguishing resistant strains from susceptible strains within the taxonomic group Lactobacillus casei/paracasei (European Food Safety Authority [EFSA], 2012).*

Finally, to unambiguously confirm that the colonies isolated on the vk-MRS plates belonged to the DG strain, we performed end-point colony PCR analysis with strain-specific primers on randomly selected colonies. The results confirmed that only those colonies with the sticky, filamentous phenotype belonged to *L. paracasei* DG. This method allowed us to precisely distinguish the DG colonies from the other fecal microorganisms and to selectively count colonies of the probiotic strain under study.

### Viable Counts of *L. paracasei* DG in the Fecal Samples of Healthy Adults

**FIGURE 3:**
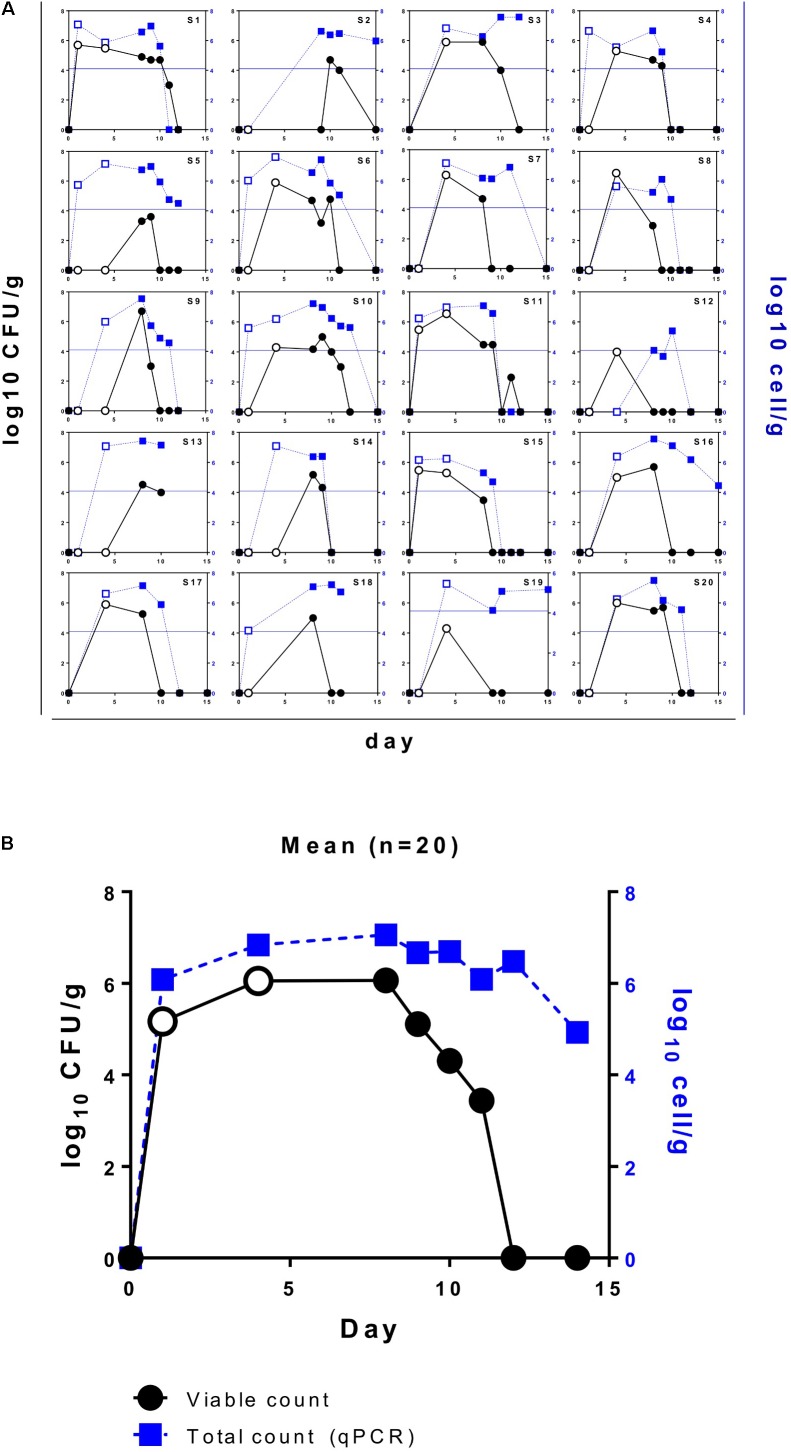
Viable (black lines and circles) and total (blue lines and squares) counts of *Lactobacillus paracasei* DG in the fecal samples of healthy adult volunteers who ingested Enterolactis in drinkable vials once daily. **(A)** Data per subject; **(B)** mean data (*n* = 20). Open symbols refer to fecal samples collected during the week of probiotic intervention.

We used the protocol based on vk-MRS medium combined with strain-specific PCR of the isolated colonies to determine viable counts of *L. paracasei* DG in the fecal samples of 20 adult volunteers over 1 week of probiotic intake and during a 1-week follow-up. Subject compliance was excellent, and all 20 subjects completed the study. Moreover, no adverse events were recorded for the entire duration of the probiotic treatment. Based on the results of strain-specific PCR (Supplementary Figure S1), 100% of the analyzed colonies with sticky, filamentous phenotypes belonged to *L. paracasei* DG, confirming that vk-MRS is a suitable medium for the selective counting of this strain. Notably, although we found wide inter-individual variability, we isolated *L. paracasei* DG from at least one fecal sample from all 20 volunteers, demonstrating that this probiotic bacterium can survive gastrointestinal transit when consumed via the formulation Enterolactis in drinkable vials. Overall, we found the highest concentration of viable *L. paracasei* DG in the fecal samples obtained between 4 and 8 days after the beginning of Enterolactis intake. The highest concentration of DG isolated from a single subject ranged between 3.6 and 6.7 log_10_ CFU per gram of feces (mean of 6.1 log_10_ CFU/g). In particular, we observed profound inter-subject variability in terms of kinetics of persistence. In fact, while from some subjects (e.g., S1) DG cells were retrieved from the first evacuation after intake of the probiotic product, from others (e.g., S2 and S9), viable DG cells were isolated only after the end of the 1-week probiotic intake period (**Figure 3**). In general, however, viable DG cells were isolated from the feces of the volunteers until 5 days after the cessation of Enterolactis intake (**Figure 3**).

### Total *L. paracasei* DG Counts in the Fecal Samples of Healthy Adults

The fecal samples collected during the 2-week trial were also used for the quantification of total *L. paracasei* DG cells by means of qPCR with strain-specific primers. As expected, the total DG counts determined via qPCR were mostly higher than the viable DG counts (**Figure 3**). Accordingly, the highest concentration of DG cells isolated from each subject, as calculated by qPCR, ranged between 5.4 and 7.6 log_10_ cells per gram of feces (mean of 7.1 log_10_ cells/g). However, while some subjects exhibited very similar total and viable counts (e.g., S1, S15, S17, and S20), others exhibited total counts that far exceeded viable counts in terms of both cell concentration and persistence (e.g., S2, S5, and S19; **Figure 3**). Overall, while the recovery of culturable DG cells was possible for up to 5 days after the intake period, the detection of DG cells by qPCR was possible for up to 7 days on average.

### Bowel Habits and DG Recovery

The Bristol Stool Chart did not reveal any significant alteration in bowel habits, and no gastrointestinal abnormalities were reported by the volunteers during the entire study. Although intestinal transit time and bowel habits could plausibly determine the differences observed among the different volunteers, the data regarding fecal types and evacuations per day did not correlate with the results of viable or total DG recovery (Supplementary Figure S2).

## Discussion

Survival of gastrointestinal transit has been listed among the criteria that a microorganism should fulfill to qualify as a probiotic (Borchers et al., 2009; Verna and Lucak, 2010). Studies of probiotic viability in humans after ingestion mostly rely on quantification in feces and are also referred to as “persistence” or “recovery” studies. The viable recovery of probiotics from feces is technically challenging because feces are microbiologically very complex, hosting thousands of different microbial species. Furthermore, probiotics mostly belong to the genera *Lactobacillus* and *Bifidobacterium*, which naturally inhabit the human gut and can, therefore, be co-isolated with the probiotic strain of interest. Reportedly, antibiotics and colony morphology have been used to address this challenge and facilitate the selective identification of colonies belonging to specific strains under study (Larsen et al., 2006; Mai et al., 2017; Poutsiaka et al., 2017). In addition, more reliable results have been obtained when molecular approaches have been combined with conventional isolation on agar plates. For instance, Tuohy et al. (2007) enumerated the *L. paracasei* strain Shirota using lactitol-LBS-vancomycin agar combined with pulsed-field gel electrophoresis to confirm colony identity. In another study, the Shirota strain was selectively quantified in feces using lactitol-lactobacillus selection-vancomycin agar plates with ELISA for confirmation of colony identity (Wang et al., 2015). Furthermore, fluorescent whole-cell hybridization was used to identify colonies of *Bifidobacterium animalis* subsp. *lactis* BB-12-like colonies grown on MRS agar supplemented with cysteine-HCl and tetracycline (Larsen et al., 2006). Molecular fingerprinting (rep-PCR, RAPD-PCR, or AP-PCR) of colonies isolated from feces has also been used to confirm strain identity (Songisepp et al., 2005; Prilassnig et al., 2007; Verdenelli et al., 2009; Pino et al., 2017).

Here, we designed an effective and reliable protocol for the selective enumeration of viable cells of *L. paracasei* DG in human feces via the exploitation of the exopolysaccharide (EPS) capsule of this bacterium (DG EPS) (Balzaretti et al., 2017). According to the analysis of the complete genome (chromosome and plasmids), *L. paracasei* DG does not have any antibiotic resistance genes (Balzaretti et al., 2015), and therefore, the modestly increased ability of the DG strain to resist certain antibiotics can be reasonably explained by the existence of the EPS capsule, which may partially impede antibiotic penetration into the cell. In addition, many strains of lactobacilli have been reported to have high natural resistance to aminoglycosides (e.g., gentamicin and kanamycin). We exploited the observed modest resistance of strain DG to certain antibiotics, which is intrinsic and is not associated with horizontally transmissible genetic elements, by adding the antibiotics vancomycin and kanamycin to the medium developed in this study to enumerate the DG strain. In addition, the DG EPS imparts a sticky, filamentous texture to the colonies, allowing easy discrimination of DG from other lactobacilli. Finally, the genetic region encoding the DG EPS has a unique DNA sequence (Balzaretti et al., 2017), therefore, permitting the design of strain-specific primers (Balzaretti et al., 2015). All the colonies with the sticky, filamentous phenotype detected on the plates were demonstrated by colony PCR with strain-specific primers as belonging to the DG strain, demonstrating that the developed protocol is suitable for the selective enumeration of strain DG.

Apparently, the literature contains contradictory reports regarding the ability of probiotic microorganisms to survive gastrointestinal transit. Previous studies, in fact, have demonstrated that the recovery of live cells of probiotic microorganisms after gastrointestinal transit in humans is poor (Hamilton-Miller et al., 1999; Temmerman et al., 2003). In a subsequent study, out of six different commercially available products, only *Escherichia coli* Nissle 1917 and *Enterococcus faecium* SF 68 were consistently detected in human feces, whereas ingested bifidobacteria and lactobacilli (including a *L. paracasei* strain) were not recovered from stool (Prilassnig et al., 2007). A higher number of studies, however, have reported the successful recovery of different probiotics from human feces after ingestion (Verdenelli et al., 2009; Derrien and van Hylckama Vlieg, 2015). Overall, the results are inconclusive, primarily because the recovery of probiotics from human feces depends on several pivotal factors: (i) the dose of the ingested live microbial cells; (ii) the intrinsic ability of the microorganism to resist chemical and physical stresses in the stomach and gut (e.g., acidity and bile salts); and (iii) the product composition in terms of excipients and/or ingredients. Accordingly, for instance, *Lactobacillus fermentum* ME-3 was retrieved from the feces of all volunteers (*n* = 16) who received the probiotic as fermented goat milk but not from volunteers who ingested the probiotic cells as gelatin-coated capsules (*n* = 12) (Songisepp et al., 2005). The results of another study showed that, compared to capsules and yogurt, cheese negatively influenced the fecal quantity of *Propionibacterium freudenreichii* subsp. *shermanii* JS and *B. animalis* subsp. *lactis* BB12 in human feces, whereas *Lactobacillus rhamnosus* GG and LC705 were not affected by the matrix (Saxelin et al., 2010). Therefore, intestinal recovery should be investigated for specific probiotic strains in precise product formulations. Nonetheless, reliable studies have been carried out only for a few well-known commercially available probiotics, such as *L. paracasei* Shirota, *L. rhamnosus* GG, and *B. animalis* subsp. *lactis* BB12. For instance, Wang et al. isolated viable cells of the Shirota strain from the feces of all volunteers (*n* = 25) after 7- and 14-day periods of consumption of a milk-based probiotic beverage (corresponding to a daily intake of approximately 10 billion CFU); however, the Shirota strain was detected in the feces of only three subjects (out of 25) 7 days after cessation of product ingestion (Wang et al., 2015). In another study, the Shirota strain was retrieved from the feces collected from 9 healthy adult volunteers after 7, 14, and 21 days of daily consumption of a fermented milk drink, corresponding to a total intake of approximately 50 billion CFU per day; 7 days after cessation of fermented milk intake, the Shirota strain was still isolated from the feces of six subjects, albeit at a much lower concentration (Tuohy et al., 2007). The longer persistence of the probiotic in a subgroup of volunteers observed in the study by Tuohy et al. (2007) than that in the REVENANT-DG trial was possibly due to the longer treatment (3 weeks) and much higher total daily intake of probiotic cells (50 billions) in the study by Tuohy et al. (2007).

More recently, the well-known probiotic strains *B. animalis* subsp. *lactis* BB12 and *L. rhamnosus* GG were successfully recovered alive from the stools of 16 out of 19 healthy volunteers who each ingested both strains together at a quantity of one billion CFU per day for 3 weeks as a powder in a sachet; the quantitative culture-based experiment, however, failed to isolate strain BB12 or GG 28 days after the end of the supplementation period (Poutsiaka et al., 2017). *L. rhamnosus* GG and *B. animalis* subsp. *lactis* BB-12, in particular, have been retrieved from the feces of healthy subjects and patients when administered with a variety of formulations, including different pharmaceutical forms and foods (Larsen et al., 2006; Ahlroos and Tynkkynen, 2009; Dommels et al., 2009; Saxelin et al., 2010; Granata et al., 2013). Nonetheless, this information is not available for most commercially available probiotics.

In our study, we examined the recovery of live *L. paracasei* DG from the GITs of healthy individuals after oral ingestion of Enterolactis, a probiotic supplement consisting of at least one billion CFU per dose of bacterial cells, suspended in a 10-ml fructose solution in drinkable vials. The intervention lasted only 1 week and was based on the intake of a single one-billion-CFU dose of probiotic cells per day; nonetheless, the intervention was effective enough to lead to the recovery of viable *L. paracasei* DG from all 20 volunteers enrolled in the study, clearly demonstrating that the Enterolactis in drinkable vials, containing at least one billion CFU, is suitable for successful delivery of probiotic cells to the human intestine.

The abovementioned studies demonstrated that the colonization of the human intestinal tract by an ingested probiotic microorganism is transient, and after the cessation of ingestion, the probiotic rapidly approaches the detection limit via kinetic mechanisms that possibly depend on the dose of the administered microbial cells. In particular, the results of our study on *L. paracasei* DG are consistent with the literature regarding the *L. paracasei* Shirota strain, the persistence of which in the guts of healthy adults was demonstrated to disappear within 1 week after cessation of probiotic intake (Tuohy et al., 2007; Wang et al., 2015).

In this study, we invited volunteers to ingest the probiotic on an empty stomach, at least 15 min before breakfast. There is no convincing information in the scientific literature to answer the question of whether probiotics should be taken with food or on an empty stomach. However, this factor could affect microbial survival in the stomach and intestine upon ingestion, and future investigations on the topic are warranted.

In conclusion, in this report, we presented the results of a comprehensive recovery study of the probiotic strain *L. paracasei* DG. Viable count experiments carried out by combining *ad hoc* culture-selective/discriminative conditions and strain-specific molecular biological protocols unambiguously demonstrated that *L. paracasei* DG can survive gastrointestinal transit in healthy adults when ingested as Enterolactis in drinkable vials, a formulation consisting of a 10-ml drinkable suspension containing no less than one billion CFU. Recovery studies to assess microbial viability after gastrointestinal transit should be a mandatory step in the characterization process of any probiotic product. Our study shows that reliable verification of microbial survival in feces can be performed in a rigorously strain-specific manner by developing enumeration protocols for viable cells based on the specific genetic and phenotypic characteristics of probiotic microorganisms of interest.

## Author Contributions

SG and WF conceived and planned the intervention trial. SG and SA developed the protocol for the strain-specific isolation of *L. paracasei* DG. SA, VT, and RK carried out the experiments. SG took the lead in writing the manuscript. All authors discussed the results and contributed to the final manuscript.

## Conflict of Interest Statement

The author WF is an employee of Sofar S.p.A., which is the company that financially supported the study. The probiotic product used in the study is commercialized by the company that financially supported the study. The remaining authors declare that the research was conducted in the absence of any commercial or financial relationships that could be construed as a potential conflict of interest.
