# Supplementary material for: Quantitative Recovery of Viable Lactobacillus paracasei CNCM I-1572 (L. casei DG®) After Gastrointestinal Passage in Healthy Adults
Source: Front Microbiol. 2018 Aug 2;9:1720. doi: 10.3389/fmicb.2018.01720 (PMC6083036; doi:10.3389/fmicb.2018.01720)

Quantitative recovery of viable *Lactobacillus paracasei* CNCM I-1572 (L. casei DG®) after gastrointestinal passage in healthy adults

Stefania Arioli, Ranjan Koirala, Valentina Taverniti, Walter Fiore, Simone Guglielmetti

Supplementary Figure 1. Confirmation of the identity of colonies by colony PCR with DG-specific primers.

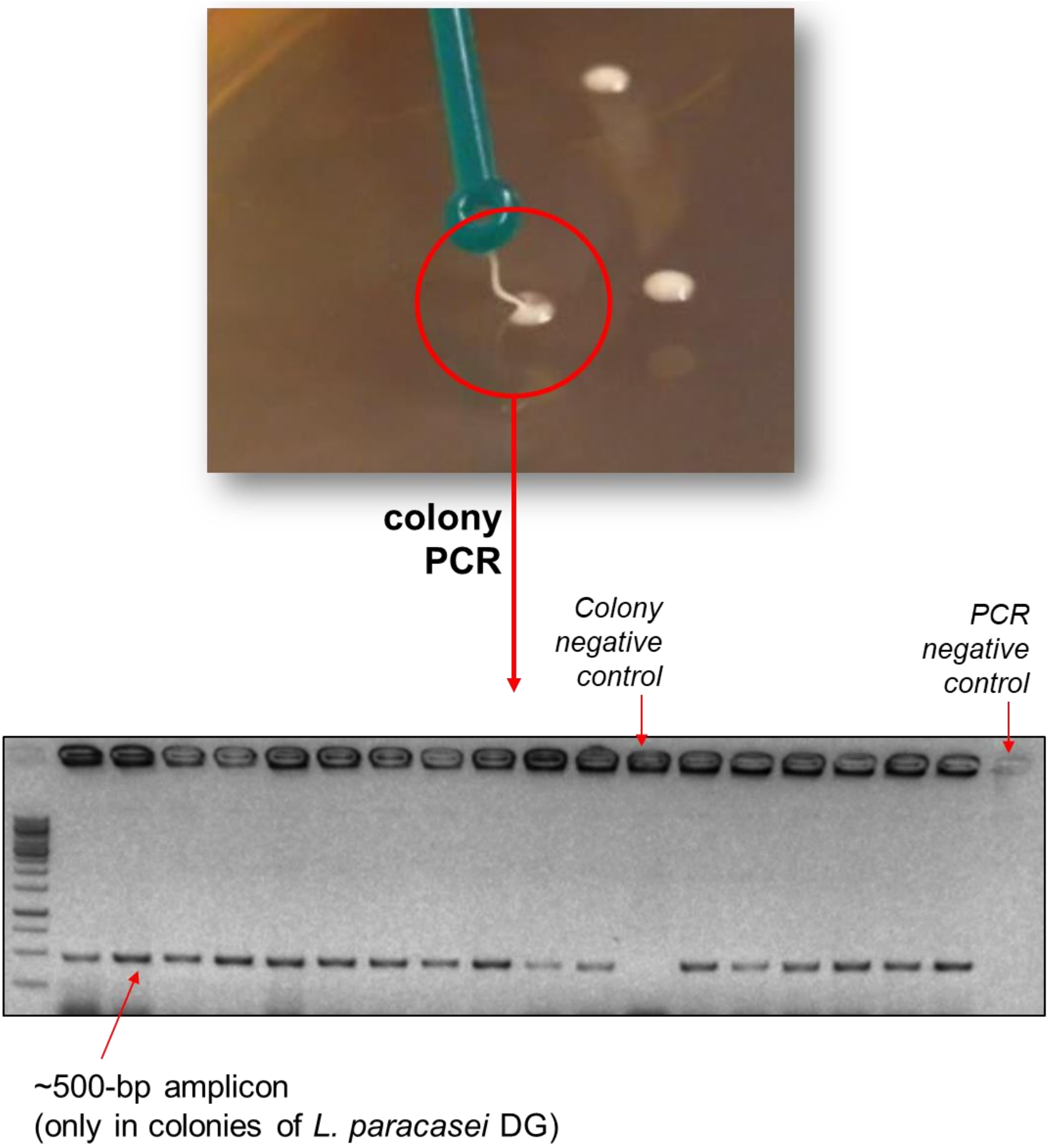

Supplementary Figure 2. Viable counts (black lines and circles) of *Lactobacillus paracasei* DG in the fecal samples, fecal type (blue columns) and evacuation per day (red squares and line) of healthy adult volunteers who ingested Enterolactis in drinkable vials (n=20). White circles refer to the viable counts of the DG strain in fecal samples collected during the week of probiotic intervention.

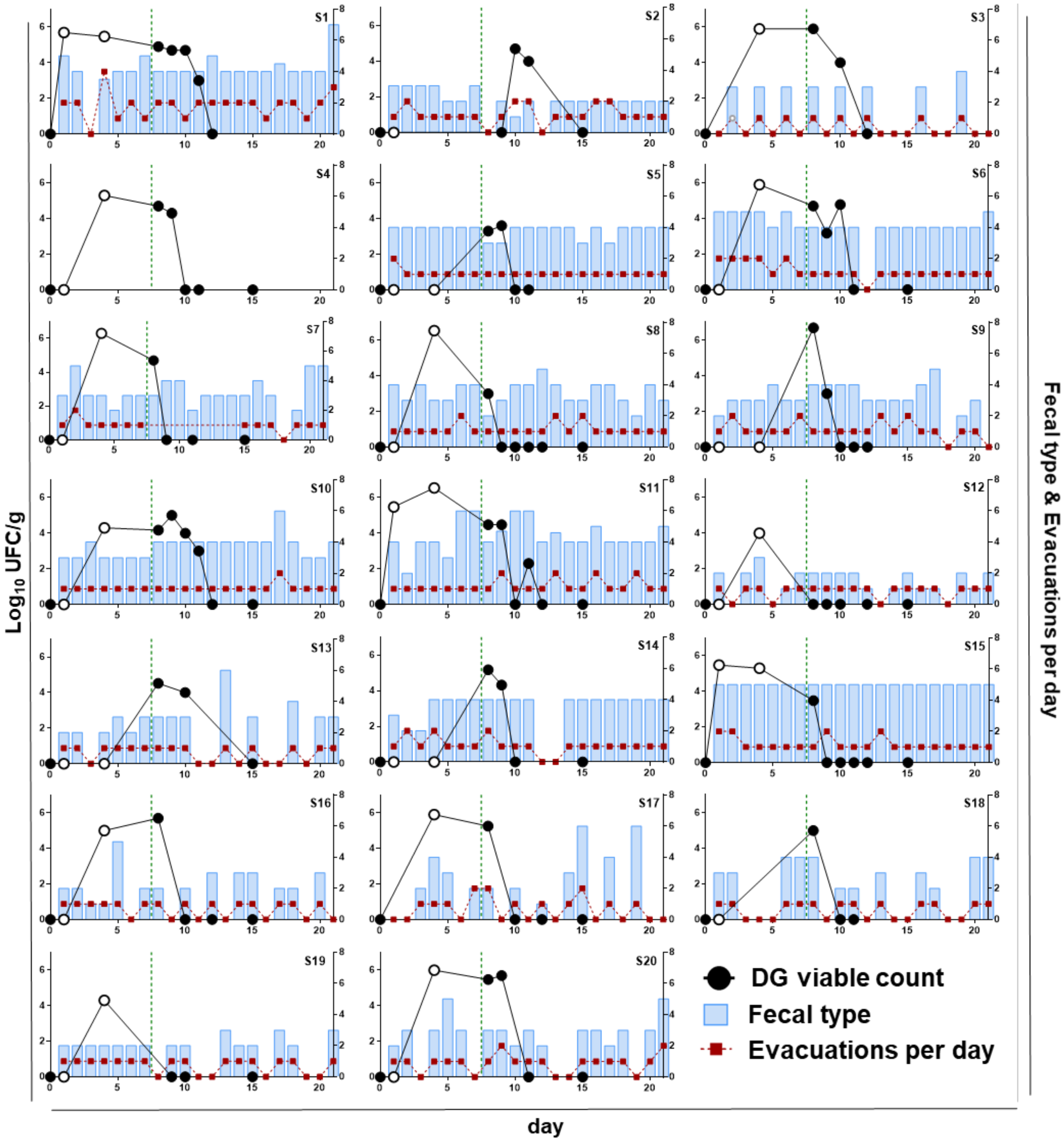

Supplement: Supplementary file 1 [file Data_Sheet_1.PDF]
