# Supplementary material for: Quantitative Recovery of Viable Lactobacillus paracasei CNCM I-1572 (L. casei DG®) After Gastrointestinal Passage in Healthy Adults
Source: Front Microbiol. 2018 Aug 2;9:1720. doi: 10.3389/fmicb.2018.01720 (PMC6083036; doi:10.3389/fmicb.2018.01720)
Supplement: Supplementary file 2 [file Presentation_1.PPTX]

## Slide 1
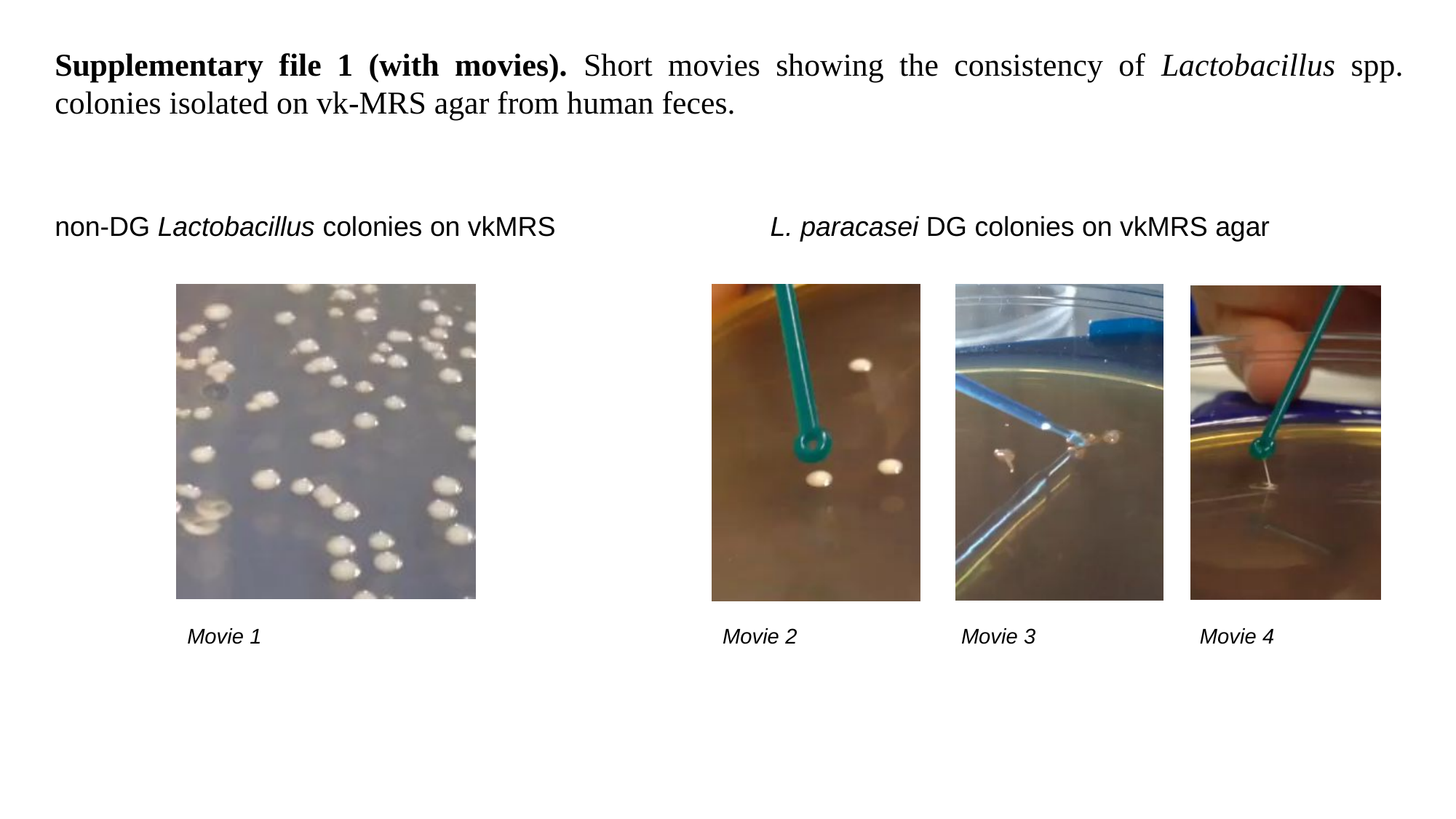

Supplementary file 1 (with movies). Short movies showing the consistency of Lactobacillus spp. colonies isolated on vk-MRS agar from human feces.
non-DG Lactobacillus colonies on vkMRS
L. paracasei DG colonies on vkMRS agar
Movie 3
Movie 4
Movie 2
Movie 1
